# Engineered Extracellular Matrices with Integrated Wireless Microactuators to Study Mechanobiology

**DOI:** 10.1002/adma.202102641

**Published:** 2021-08-07

**Authors:** Fazil E. Uslu, Christopher D. Davidson, Erik Mailand, Nikolaos Bouklas, Brendon M. Baker, Mahmut Selman Sakar

**Affiliations:** ^1^ Institute of Mechanical Engineering and Institute of Bioengineering Ecole Polytechnique Fédérale de Lausanne Lausanne CH‐1015 Switzerland; ^2^ Department of Biomedical Engineering University of Michigan Ann Arbor MI 48109 USA; ^3^ Sibley School of Mechanical and Aerospace Engineering Cornell University Ithaca NY 14850 USA

**Keywords:** extracellular matrix, finite‐element modeling, mechanobiology, micromanipulation, robotics

## Abstract

Mechanobiology explores how forces regulate cell behaviors and what molecular machinery are responsible for the sensing, transduction, and modulation of mechanical cues. To this end, probing of cells cultured on planar substrates has served as a primary experimental setting for many decades. However, native extracellular matrices (ECMs) consist of fibrous protein assemblies where the physical properties spanning from the individual fiber to the network architecture can influence the transmission of forces to and from the cells. Here, a robotic manipulation platform that allows wireless, localized, and programmable deformation of an engineered fibrous ECM is introduced. A finite‐element‐based digital twin of the fiber network calibrated against measured local and global parameters enables the calculation of deformations and stresses generated by different magnetic actuation schemes across a range of network properties. Physiologically relevant mechanical forces are applied to cells cultured on the fiber network, statically or dynamically, revealing insights into the effects of matrix‐borne forces and deformations as well as force‐mediated matrix remodeling on cell migration and intracellular signaling. These capabilities are not matched by any existing approach, and this versatile platform has the potential to uncover fundamental mechanisms of mechanobiology in settings with greater relevance to living tissues.

## Graphical abstract

An in vitro biomimetic platform that provides independent control over geometry, mechanics, and structure over extracellular matrices is introduced. Remote application of forces using robotically controlled magnetic microactuators triggers native mechanobiology responses including intracellular signaling and directed migration. A digital twin complements the platform by reporting stresses generated upon actuation.

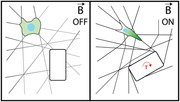

## 1 Introduction

Many cells reside within complex 3D ECMs consisting of an interconnected network of micrometer‐thick fibers. The filamentous architecture of the ECM and resulting mechanical properties have a direct influence on numerous biological processes, including those related to homeostasis, pathology, and regeneration.^[^ ^1^, ^2^, ^3^, ^4^ ^]^ Throughout these processes, cells probe and respond to the mechanical properties of the ECM using contractile forces and assemblies of mechanosensitive proteins.^[^ ^5^, ^6^, ^7^, ^8^ ^]^ Furthermore, discrete network architecture and nonlinear mechanics generate a spatially inhomogeneous and temporally dynamic reaction to the action of external and internal loads, including hydrostatic pressure, shear stress, and contractility of neighboring or distant cells.^[^ ^9^, ^10^ ^]^ To dissect all of this complexity, fibrous matrices engineered from natural or synthetic materials have served as in vitro mechanobiology platforms. These platforms recapitulate the structural and mechanical properties of native tissue ECMs.^[^ ^11^ ^]^ In particular, electrospun dextran fiber networks are particularly suited for this purpose given their tunable architecture (density, alignment, diameter), mechanics (stiffness, degradability), and surface chemistry.^[^ ^12^ ^]^ Experiments using this tunable material platform have revealed that cells probe and respond to mechanics in fibrous matrices by recruiting nearby fibers, a mechanism that has profound impact on cell migration, proliferation, and the assembly of multicellular structures such as vascular networks.^[^ ^12^, ^13^, ^14^ ^]^

Controlled mechanical micromanipulation of cells has played an instrumental role in the discovery of proteins and signaling pathways that sense mechanical signals and translate them into biochemical responses.^[^ ^15^ ^]^ Adherent and suspended cells have been mechanically stimulated using magnetic and optical tweezers, aspiration micropipettes, microfluidics, and indentation probes.^[^ ^16^, ^17^, ^18^, ^19^, ^20^, ^21^, ^22^, ^23^, ^24^, ^25^, ^26^, ^27^ ^]^ Alternatively, tensile forces can be applied to cultured cells on mechanically active substrates such as stretchable polymer sheets, actuated pillar arrays and photothermal hydrogels.^[^ ^28^, ^29^, ^30^, ^31^, ^32^, ^33^, ^34^, ^35^, ^36^, ^37^, ^38^ ^]^ Similar manipulation techniques have been explored to characterize the mechanical properties of tissues. Magnetically and thermally responsive probes have been encapsulated inside embryonic tissues or reconstituted collagen matrices for the characterization of tissue stiffness.^[^ ^39^, ^40^, ^41^, ^42^ ^]^ A spatiotemporally controlled actuation paradigm capable of generating physiologically relevant forces within engineered fibrillar microenvironments could open new avenues to study the mechanics of cell‐ECM interactions, enabling insight into how these interactions lead to the emergence of self‐organization during development or aberrant re‐organization in the course of conditions such as fibrosis and metastasis.

**Figure 1:**
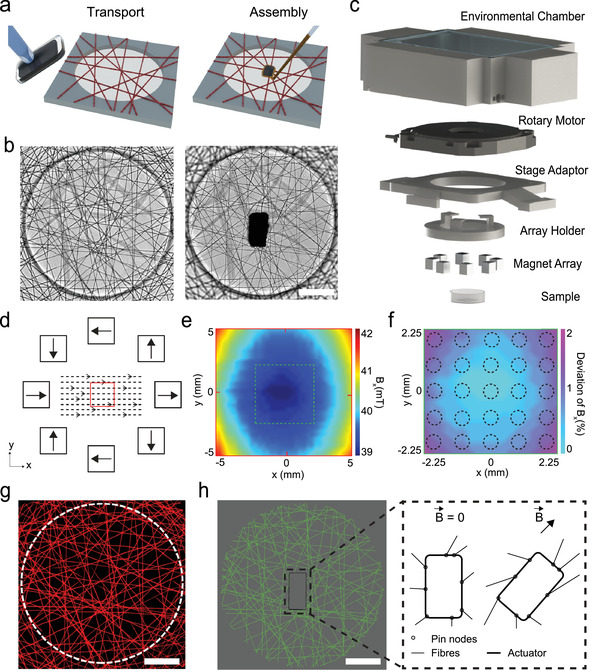
Experimental platform and the digital twin. a) Schematics illustrating the positioning and assembly of magnetic microactuators (black block). The red beams represent DexMA fibers. DexMA is electrospun onto microfabricated substrates (shown in gray) to define networks of suspended fibers. b) Phase‐contrast images of a fiber network before (left) and after (right) the incorporation of a µ‐actuator. c) 3D drawing of the magnetic manipulation system. The uniform magnetic field is generated using a magnet array that is embedded into an array holder. The orientation of the µ‐actuator is determined by the magnet array that is controlled by a rotary piezo motor. Environmental chamber maintains physiological temperature, pH and humidity inside the workspace. d) Schematics showing the arrangement of the magnets within the array and the distribution of the magnetic field at the plane of the fiber networks. Red square denotes the area in which the fiber networks are located. e) Heat map of the recorded magnetic flux density inside the workspace. Highlighted area (dashed lines) contains a 5 × 5 grid of fiber networks from which the data is collected. f) Measured deviation in the x component of the magnetic flux density proves the homogeneity of the magnetic field. g) A high‐resolution fluorescence image of the fiber network that is used to generate the digital twin. h) A one‐to‐one digital replicate of the fiber network that is shown in (g). The discrete fiber network model is used to simulate the deformation according to the actuation signal. Pin nodes are generated between the fibers and the µ‐actuator that are free to rotate. Scale bars: 100 µm.

The study of force transmission through ECM has certain technical specifications. On one hand, to recapitulate the fiber recruitment process that leads to multicellular organization, relatively large local deformations must be generated and sustained over long periods of time, lasting hours or even days. On the other hand, cells respond to dynamic mechanical stimulation within microseconds to minutes through calcium signaling, changes in focal adhesions or cell‐cell junctions, remodeling of the actomyosin cytoskeleton, and nuclear translocation of transcriptional regulators such as Yes‐associated protein (YAP) and myocardin related transcription factor‐A (MRTF‐A).^[^ ^43^, ^44^, ^45^, ^46^, ^47^ ^]^ Here, we present a technology to mechanically interface with cells cultured on engineered fibrous matrices while simultaneously monitoring the cellular responses and force‐mediated changes in network architecture in real‐time. The technology is based on magnetically responsive microactuators (µ‐actuators) that are integrated into synthetic fibrous matrices with cell‐scale precision using robotic micromanipulation (**Figure** **1**a). We have also developed a digital twin of the experiment using imaging, computer vision, multiscale mechanical characterization, and finite element modeling of discrete fiber networks to enable virtual testing of mechanical actuation schemes and quantification of mechanical stresses from empirical data.

## 2 Results

Transport and assembly of the µ‐actuators were performed using a dexterous motorized micromanipulation system and two distinct end effectors (Figure S1, Supporting Information). Both tools were pulled and forged from glass microcapillaries according to the size and geometry of the µ‐actuator. The first end‐effector is an aspiration micropipette connected to a microfluidic pressure system that provided dexterous transport and precise positioning of the µ‐actuators at desired locations on the fiber network. The second end‐effector consisted of a microinjection pipette connected to a pneumatic picopump for precise delivery of a biocompatible adhesive around the µ‐actuator. A digital micromirror device initiated the spatially controlled photopolymerization of the glue, completing the actuator integration procedure. The glue embedded the µ‐actuator into the fibrous matrix for the effective transmission of forces. With this methodology, µ‐actuator(s) could be assembled at prescribed locations at defined orientations (Figure 1b and Figure S2, Supporting Information). We did not measure any slack on the fiber networks due to the weight of the actuator or glue (Figure S3, Supporting Information).

Matrices were generated from electrospinning methacrylated dextran (DexMA), which produces fibers that are resistant to non‐specific protein adsorption. Fiber networks were deposited on a surface‐functionalized array of microfabricated PDMS elastomer wells such that fibers were suspended across the wells. Fibers that resided outside the wells firmly attached to the elastomer surface, therefore, rigid boundaries were defined by the well perimeter. Networks were placed in a controlled humidity environment and provided sufficient moisture before light exposure to fuse or "weld" all juxtaposed fibers. The platform allows execution of tens of experiments in parallel with user‐defined initial conditions. Once the integration of the µ‐actuator was completed, the substrate was submerged in culture media. The magnetic manipulation system consists of an array of eight permanent magnets spatially arranged to maximize the magnetic torque (Figure 1c and Figure S4, Supporting Information). A custom‐design chamber that sits around the actuator ensures maintenance of physiological conditions. The magnetic field is uniform over 4.5 × 4.5 mm^2^ area on the plane that coincides with the sample, thus, the system only applies torque to magnetic structures that are within the workspace (Figure 1d–f). We recorded the motion of µ‐actuators located in the outermost wells during rotation of the external field and verified that the actuators did not move toward the permanent magnets. The magnetic field strength was kept at 40 mT in the center of the chamber while the system modulated the orientation of the magnetic field by precisely rotating the array using a piezoelectric motor with nanometric resolution. As a result, the system could hold the actuator at the desired orientation indefinitely during which the motor was turned off, enabling long‐term time‐lapse experiments. At the same time, the system operates at an angular velocity ranging from 0.0001 to 9.5 rad s^−1^, providing tunable strain rates with high resolution for the application of static or cyclic mechanical loading.

We generated a digital twin of the physical experiment by computationally reconstructing the fiber topology and mechanics, and the motion of the actuator. A high‐resolution laser scanning confocal image of the network informed the reconstruction process (Figure 1g). We assumed that all fibers were on the same plane and an apparent intersection of fibers on the 2D projection of the volumetric image stack constituted a permanent interfiber crosslink. With these assumptions, the image was processed to generate a list of nodes and links, which together represented the network topology. The links showed how nodes were connected to each other in each well, including the connections to the wall, which was segmented according to the arrangement of fibers crossing the barrier. The boundary conditions at the wall positions were considered to be fixed. A computational model was then created by interconnecting linearly elastic Timoshenko beam elements conforming with the topology of links (Figure 1h), while accounting for finite deformations in 3D space. The average diameter of the fibers was measured as 1.8 µm from microscopey images and we set the diameter of all the beam elements as such in the model. Experimentally, the matrix was imaged after integrating the µ‐actuator, to define its exact position and orientation within the digital twin. A new set of nodes was created around the contour of the µ‐actuator at connection points to the fibers, and a rigid plate of identical geometry to the µ‐actuator was pinned to the network at those points. Finally, actuation was modelled by imposing either displacement or torque of the rigid plate.

**Figure 2:**
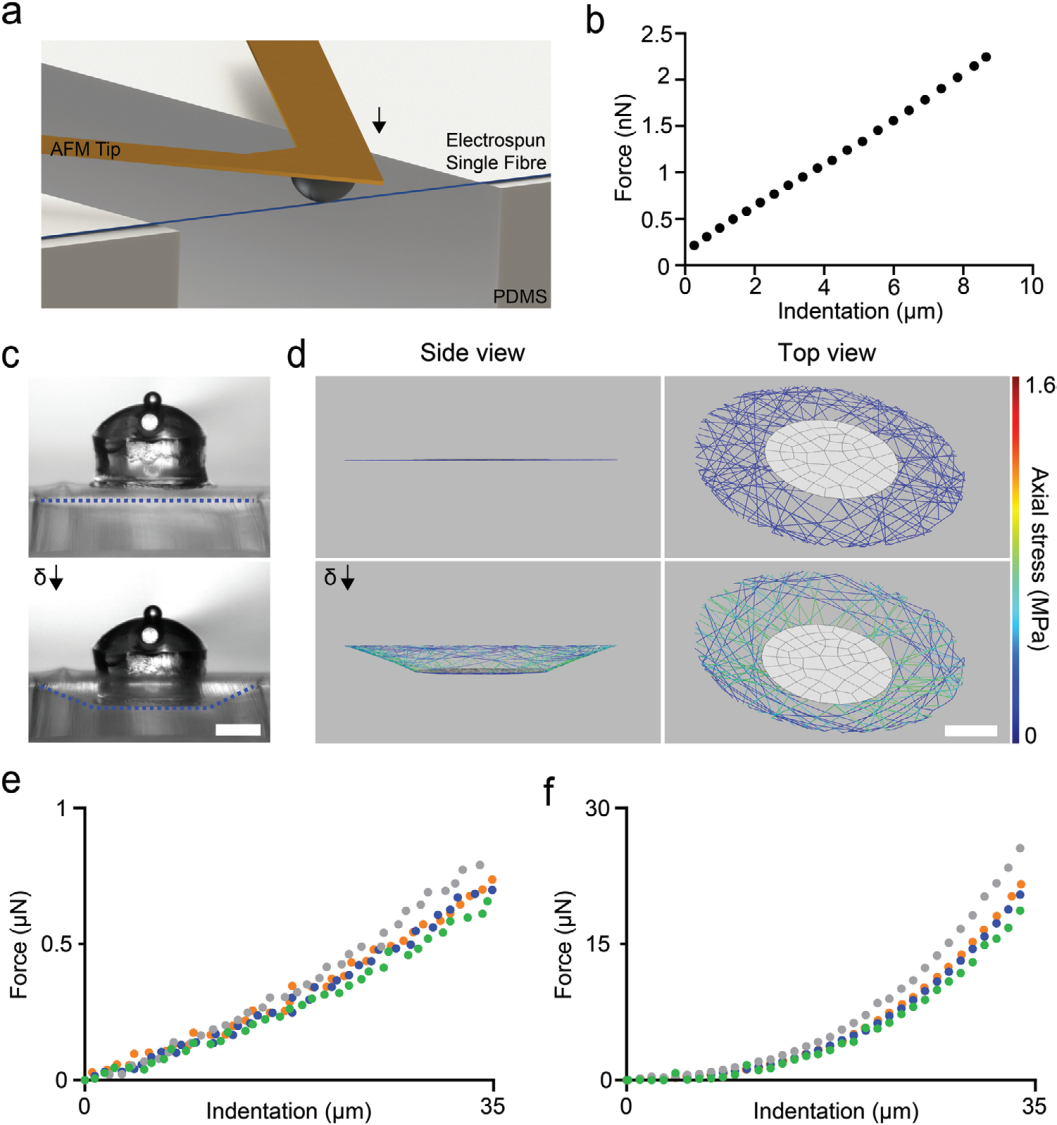
Mechanical characterization and model validation. a) Three‐point bending tests are performed on single fibers using AFM. b) A representative force versus deflection curve. The Young's modulus of the fibers is calculated from these measurements. c) Microscopy images from a bulk indentation experiment. The cylinder with a dome‐shaped cap is the indenter and the dashed lines (blue) highlight the deformation of the fiber network. d) The indentation of the digital twin of the sample shown in (c) with identical conditions (i.e., indenter size and indentation depth). e) Empirical and f) simulated force values from the indentation trials for four different fiber networks in their hydrated state. Scale bars: 100 µm.

The digital twin consists of a constrained network of beams that is a one‐to‐one topological replica of the experimental specimen. We considered only a single layer of fibers in our virtual reconstruction while matrices had three to five layers of fibers due to the layering inherent to electrospinning—however, matrices were briefly exposed to humidity to weld points of interfiber contact, as described previously.^[^ ^12^ ^]^ As such, all apparent intersections of fibers from a 2D projection of the network were considered to be permanently linked. With the use of correct constitutive equations appropriate for the range of local fiber deformations, the computational model is expected to accurately estimate the mechanical stresses, effectively realizing traction force microscopy on a fibrous substrate. Nanoindentation of hydrated single DexMA fibers using atomic force microscopy (AFM) showed that constitutive behavior of individual fibers can be captured through isotropic linear elasticity with a Young's modulus of 22 ± 5.53 MPa (**Figure** **2**a,b). The simulations were run based on the empirical mean value of the Young's modulus while Poisson's ratio was taken as 0.49, assuming incompressibility of the fibers. To experimentally validate the in silico force calculations, we performed microindentation of fiber networks (Figure 2c and Movie S1, Supporting Information). In tandem, the digital copy of the discrete network was mechanically loaded using a model of the rigid indenter with the same dimensions, assuming no friction at the contact area (Figure 2d). The fiber network can be approximated as a thin linearly elastic perforated membrane at the continuum level, and as such, geometric nonlinearities are expected to arise when the specimen is significantly indented. The nonlinear response was evident in the shape of the force‐indentation curves (Figure 2e,f).

The fibers swelled upon hydration, where the diameter increased by 60% from 1.12 ± 0.15 to 1.8 ± 0.2 µm. The associated volumetric expansion has two important consequences for the network mechanics: a reduction in the Young's modulus of the fibers and fiber buckling due to the constraints around the network (Figure S5, Supporting Information). We performed additional indentation tests in air to quantify the effect of fiber swelling on network mechanics (Figure S6, Supporting Information). The measured forces were two orders of magnitude higher than the values recorded on hydrated samples (Figure 2e). This discrepancy may arise from two major differences between the physical matrix and theoretical model. First, we created the computational model based on the non‐swollen state of the network (capturing network connectivity and fiber topology) due to the difficulty of modelling buckled fibers. A network with links that do not bear loads is expected to be softer compared to a network in which all the fibers are tight at the initial state. In addition, the connectivity of the network is higher in the model because we projected multiple layers of fibers into a single plane and assumed that all apparent intersections of fibers are welded crosslinks. As expected, the simulated network stiffness was higher than the stiffness measured from hydrated samples (Figure 2f).

We computed the magnetic torque acting on the soft magnetic actuator by modeling its shape as ellipsoid. The magnetic torque that tends to align the long dimension of the body with the applied field is given by:

(1) $$\begin{array}{c} T\;=\mu_{0}\nu \;\left(M\;\times \;H\right) \end{array}$$

where the body lies in an external field with a value **H** at the body's center of mass and the field magnetizes the body to a magnetization **M**. The volume of the magnetic material is denoted by ν and the permeability of free space is μ_0_ = 4π × 10^−7^ T m A^−1^. At fields low enough such that |**M**| < *m*_s_, the magnitude of the torque can be computed as^[^ ^48^ ^]^

(2) $$\begin{array}{c} \left|T\right|=\frac{\mu_{0}\nu \left|n_{r}-n_{a}\right|}{2n_{a}n_{r}}\;{\left|H\right|}^{2}\sin\left(2\theta \right) \end{array}$$

where *n*_a_ and *n*_r_ are the demagnetization factors, and θ is the angle between **H** and the axis of symmetry. The maximum torque applied by a µ‐actuator, that is 100 µm long and 50 µm wide, is calculated as |**T**|_max_ = 1.06 × 10^−9^ N m. When we applied |**T**|_max_ in the digital twin of a particular fiber network, the actuator rotated 2.75° instead of the empirically recorded rotation of 88° (Figure S7, Supporting Information). As described above, this discrepancy is due to the overestimation of network stiffness in our model. As indicated by the indentation data (Figure 2e,f), the force calculated by the computational model was higher than the measured value for the actual samples. We recapitulated the experimentally recorded deformation by scaling the input torque to the same extent, i.e., by taking |**T**|_max_ = 3.15 × 10^−8^ N m (Figure S7, Supporting Information).

**Figure 3:**
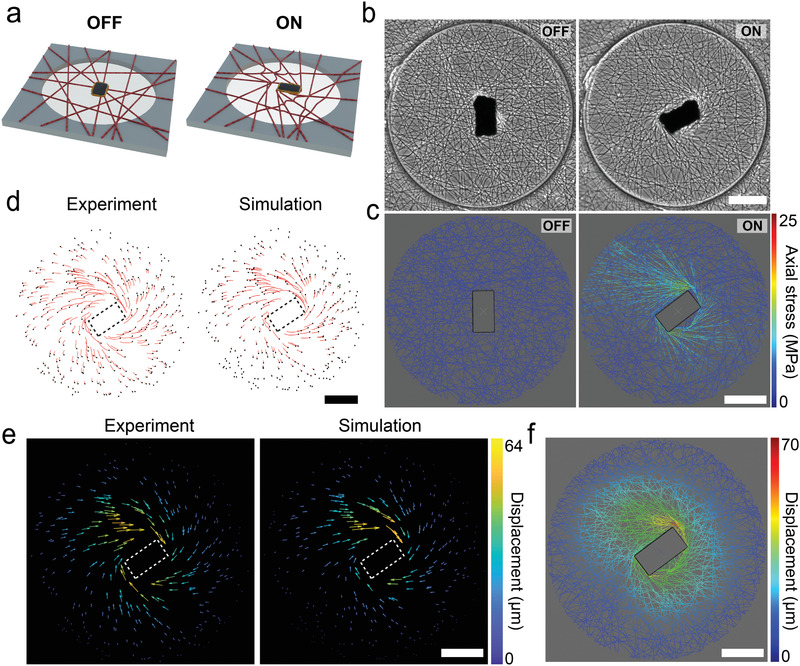
Experimental and computational investigation of the actuation paradigm. a) As soon as the network is placed within the magnetic manipulation system, a torque clamp is activated for the µ‐actuator. Any deviation on the orientation of the µ‐actuator would generate a restoring magnetic torque. The orientation of the µ‐actuator is dynamically modulated to apply a local deformation to the fiber network. b) Phase‐contrast images showing the operation of the µ‐actuator. The magnetic field is rotated by 90° from the equilibrium configuration. c) A digital twin of the fiber network shown in (b) is manipulated to the same degree. The simulation results report the stress on the fibers along with the strain. d) The deformation is quantified by tracking fluorescent beads randomly distributed on the fiber network (left). The trajectories of points at the positions of the fluorescent beads are calculated by the computational model (right). e) The motion of the nodes introduced in (d) are displayed as a vector field for the experiment (left) and the digital twin (right). f) The displacement of all the nodes within the network are calculated using the digital twin. Scale bars: 100 µm.

The samples were placed inside the workspace of the magnetic manipulation system such that the µ‐actuators were aligned along the direction of the external homogenous magnetic field (**Figure** **3**a). This initial configuration corresponding to a state of zero magnetic torque was considered an equilibrium state. The µ‐actuator synchronously rotates with the external field to minimize the magnetic dipole interactions and lower the energy (Movie S2, Supporting Information). In theory, there exists a field rotation frequency, known as a step‐out frequency, above which the applied torque is not strong enough to keep the actuators synchronized with the field. Our µ‐actuator followed the driving field even at the maximum speed of the system (Movie S3, Supporting Information)—thus, step‐out was not a concern in these experiments. The fiber network is elastic, applying forces to resist the rotation of the µ‐actuator. This restoring spring force scales with angular displacement and there is a theoretical maximum where the torque of the µ‐actuator is maximally resisted by the matrix. For the chosen physical properties of the fibers, µ‐actuators, and magnetic manipulation system, this angle was recorded as 90° ± 6°. Decreasing the density or stiffness of the fibers and increasing the shape anisotropy or volume of the µ‐actuator could result in a higher maximum displacement angle.

Rotation of the µ‐actuator resulted in spatially heterogeneous forces in the network, with tensile or compressive mechanical stresses dependent on the fiber orientation and local connectivity. As a result, some of the fibers were stretched while others buckled due to compression (Figure S8, Supporting Information). Fibers attached to the left‐top and right‐bottom corners of the µ‐actuator became loaded in tension, which led to formation of aligned fiber bundles that were under tension (Figure 3b). The overall deformation of the network was measured using fluorescent microbeads encapsulated inside the DexMA fibers (Movie S4, Supporting Information). We applied the same mechanical load to the digital twin by rotating the rigid plate in the model to the equilibrium orientation of the µ‐actuator (Figure 3c). We defined points in the computational model that corresponded to the exact empirical positions of the fluorescent microbeads to compare the displacement of the fibers during the actuation. Figure 3d shows representative plots displaying the trajectories of the beads. The particle tracking velocimetry data is also shown as displacement vectors, drawn from the initial to the final position of the marker points (Figure 3e). The results showed that the digital twin accurately predicted the deformation of fibers in response to the motion of the µ‐actuator. Notably, reeling of the surrounding fibers due to the rotation of the µ‐actuator recapitulated the fiber recruitment resulting from cell generated forces. A single endothelial cell pulls the adjacent fibers up to 50 µm before making new connections or migrating forward (Figure S9, Supporting Information) while the rotation of the µ‐actuator by 90° resulted in 70 µm displacement of the nearby fibers. Importantly, the digital twin can provide high‐resolution information on the deformation along with stresses and strains of the individual fibers (Figure 3f).

The computational framework could be used to interrogate the combinatorial effects of network topology and actuation protocol (e.g., the number, distribution, size, and shape of actuators) on the deformation of the network. As a proof‐of‐concept example, we fixed the configuration of the actuator and simulated the deformation for two extreme network topologies, i.e., fibers aligned uniaxially and patterned as a regular grid. The simulation results clearly showed that, depending on the network topology, a cell might experience very different mechanical loads for the same input torque and µ‐actuator configuration (Figure S10, Supporting Information). As a further inquiry on the importance of network topology, we removed only a few connections from a representative network while keeping everything else intact. The stress changed significantly at certain locations upon actuation, corroborating with the previous results (Figure S11, Supporting Information). Taken together, these demonstrations showed that replicating the exact topology of the network in computational simulations is essential for the accurate prediction of deformation and associated stress in discrete fibrous substrates.

**Figure 4:**
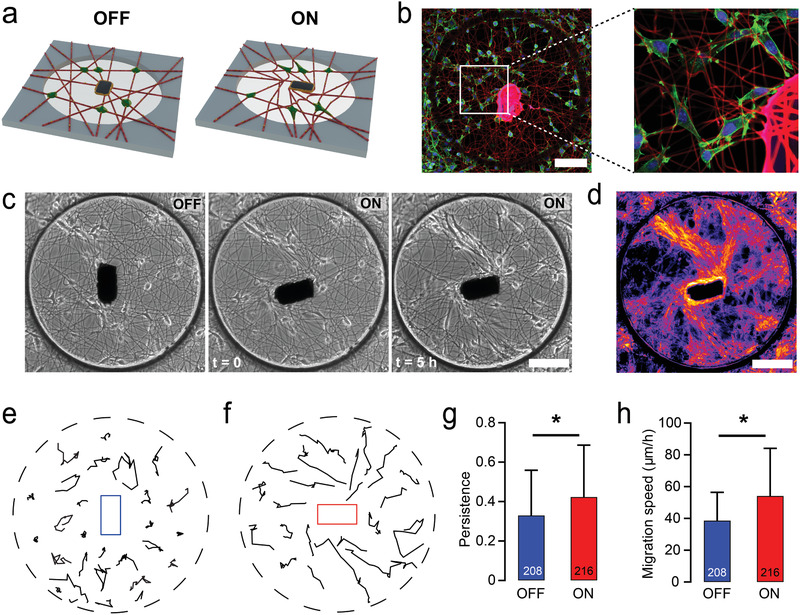
Local matrix alignment by the µ‐actuator influences cell migration speed, directionality, and persistence. a) Schematics illustrating the local actuation of the fiber network seeded with fibroblasts (green). b) Fibroblasts adhere to the RGD‐coupled fibers, spread, and migrate. Cells were counter‐stained for F‐actin (green) and nuclei (blue) with phalloidin and Hoechst 33342, respectively. The glue polymerized around the µ‐actuator shows autofluorescence in the red channel. c) Temporal sequence of phase‐contrast images shows the increasing density of fibroblasts migrating along the aligned fibers. d) Heat map of the overlay of microscopy images taken every 15 min for 6 h highlight the cell trajectories. e,f) Representative fibroblast migration tracks over a 6 h time course on non‐actuated (e) and actuated (f) fiber networks. g,h) Key characteristics of cell migration such as persistence (g) and migration speed (h) increase with the local actuation. All data presented as mean s.d. and * indicates a statistically significant comparison with *p* < 0.05 (two‐way analysis of variance). Scale bars: 100 µm.

We started our biological investigation by asking the following question: Can we influence cell migration in our platform with the strain generated by the µ‐actuator? To this end, we seeded fibroblasts on fibers that were functionalized with cell‐adhesive peptide RGDas as illustrated in **Figure** **4**a. 3T3 fibroblasts adopted a spindle shape possessing thin, elongated processes that terminated in branched protrusions, resembling their morphology on type I collagen matrices (Figure 4b). The system can be operated under a torque clamp without consuming power. When the adherent cells changed the orientation of the µ‐actuator by pulling on the fibers at the equilibrium state, a magnetic torque whose magnitude depended on the misalignment angle acted to restore the orientation of the µ‐actuator (Movie S5, Supporting Information). While maintaining the initial orientation, the µ‐actuator occasionally translated along with the connecting fibers. This experiment showed that the forces applied by the magnetic system to the network is comparable to the forces applied by the cells.

Physical features of the ECM such as rigidity and geometry influence cell morphology, polarization, and cell motility.^[^ ^49^, ^50^ ^]^ This process can emerge as a result of the local deformations and remodeling of the fibrillar ECM. We mechanically loaded the networks by rotating the magnetic field 90° in clockwise direction at 0.1 rad s^−1^. Fibroblasts seeded in the vicinity of the µ‐actuator moved to the aligned fibers within 2 h and constrained their motion along these lines of tension during the remainder of imaging (Figure 4c and Movie S6, Supporting Information). Overlaying phase‐contrast images of the samples highlighted deterministic trajectories that followed the same multicellular activity pattern in all the actuated wells (Figure 4d). Increasing local fiber alignment led cells to adopt an elongated uniaxial morphology and migrate with enhanced speed and persistence compared to the control case where the fiber networks were left in the equilibrium state (Figure 4e–h). We have recently shown that cells stretch matrix fibers to store elastic energy and subsequent adhesion failure at the cell's trailing edge can trigger a sudden matrix recoil and rapid cell translocation.^[^ ^14^ ^]^ This distinct mode of migration, which we termed slingshot migration, was displayed more frequently on aligned matrices. We recorded a 21.9% higher occurrence of slingshot migration on actuated matrices (see Movie S7, Supporting Information), supporting the critical role of aligned fibers and matrix‐borne forces in this phenomenon.

**Figure 5:**
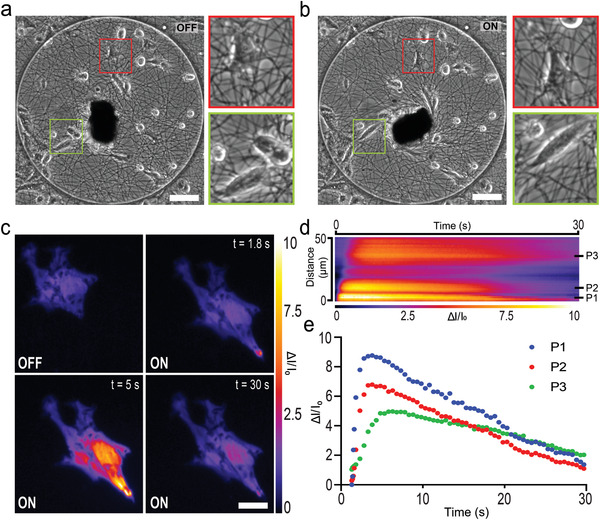
Mechanical loading of epithelial cells through fibers activates stretch‐activated ion channels. a) Bright‐field image of a fiber network seeded with epithelial cells. Two single cells are magnified (red and green boxes) to clearly show the effect of actuation. b) The network shown in (a) is actuated as the magnetic field is rotated by 90° from the equilibrium configuration. Insets show the tensile loading of the cells. Scale bars, 100 µm. c) Time‐lapse images of Ca^2+^ indicator shows the initiation, propagation and decay of the intracellular signal. Scale bar, 20 µm. d) Kymograph capturing the spatiotemporal propagation of the Ca^2+^ signal on the central line of the cell presented in (c). The line starts from the tip of the cell (bottom right of the image) and ends at the other end of the cell. e) The normalized intensity of the fluorescence signals are recorded from three different points inside the cell. Locations of these points are indicated on the kymograph shown in (d).

The rotation of the µ‐actuator not only modifies ECM alignment but also presents active mechanical cues by stretching constituent cells. Cells can detect and transduce mechanical forces into biomechanical signals using a variety of mechanosensitive proteins. Of particular interest, stretch‐activated ion channels respond to membrane tension by altering their conformation between an opened and closed state, facilitating mechanically gated ion flux into cells.^[^ ^51^ ^]^ Among different anions and cations, intracellular signaling initiated by calcium (Ca^2+^) passage has been shown to play a key role in mechanotransduction of non‐muscle cells.^[^ ^52^, ^53^, ^54^ ^]^ We used fluorescence video microscopy and a Ca^2+^ indicator to monitor signal transduction in cells during the course of actuation. Epithelial cells were cultured on the substrate at a relatively low density to ensure that forces were transmitted through the fibers and not through cadherin junctions. Cells that reside along the direction of tension were clearly stretched in the course of actuator rotation (**Figure** **5**a,b). We recorded up to 10‐fold increase in fluorescence intensity of the Ca^2+^ indicator above the baseline value within 1 to 5 s after force application which returned to the baseline within 20 to 30 s. Notably, the signal initiated at the pole of the cell nearest the µ‐actuator, and propagated through the rest of the cell body (Figure 5c–e and Movie S8, Supporting Information). We postulate that the spatiotemporal propagation of the signal was due to the release of Ca^2+^ from the intracellular stores in response to the influx of extracellular Ca^2+^ across the plasma membrane stemming from matrix stretch.

## 3 Discussion

Actuation based on the application of magnetic torque has several advantages over alternative techniques. Optomechanical actuation, which is particularly appealing considering the spatial and temporal resolution of laser illumination,^[^ ^34^, ^37^, ^38^, ^55^ ^]^ is limited in throughput because simultaneous illumination of multiple distributed actuators is not possible in existing systems. Furthermore, continuous illumination is required in order to apply forces, which raises issues regarding local heating and power consumption. Our technology not only addresses these issues, but also generates significantly higher deformation on matrices through rotation and reeling of fibers (see Table S1, Supporting Information). To be able to generate higher maximum deformation with the same magnetic manipulation system, the aspect ratio of the µ‐actuator could be increased. If the experiment requires the generation of the same level of deformation using a smaller µ‐actuator, the actuator could be manufactured from a material with higher magnetization.

The simulated value of the indentation force was an order of magnitude higher compared to the measurements made in a hydrated state. We made three basic assumptions in the construction of the model: i) the loading state does not change after the swelling process, ii) there is only one layer of fibers, and iii) all the nodes correspond to stable fiber connections. As shown by the experiments, swelling of fibers led to local buckling due to boundary conditions. In addition, although we applied a process to weld fiber intersections prior to hydration, we recorded that fibers occasionally slid with respect to each other at contact points during experiments. Connectivity has a significant effect on the apparent elastic modulus of a network of randomly aligned fibers.^[^ ^56^ ^]^ Overestimation of network connectivity and omitting hydration‐driven mechanical instabilities led to a stiffer virtual twin of the network at the continuum level compared to the actual specimen. Each sample consisted of 5–10 layers of fibers, which was defined by the duration of fiber deposition during the electrospinning process (Figure S12, Supporting Information). As such, the cells were semi‐embedded in the fibrous matrix, with cell adhesions distributed across multiple layers of fibers but with cell spreading taking place largely in the *x–y* plane. More realistic models of the networks can be generated by: i) introducing volumetric fiber swelling to capture the buckling instabilities upon hydration, ii) using a realistic 3D model of the network that is obtained via higher resolution volumetric reconstruction, and iii) incorporating fibers that come in contact with each other without being rigidly bonded. We modeled fibers as 3D structures, thus, within the existing framework, we can increase the number of layers in the network and calculate deformation and stress in a 3D space. While this change may make the model more realistic, the computation time will increase significantly.

Ca^2+^ imaging experiments showed that only the cells adhering to fibers directly connected to the corners of the µ‐actuator experienced stretch‐activated Ca^2+^ flux. The rest of the fibers within the network also bore stress, but potentially not at high enough levels to open stretch‐activated ion channels. In addition to the amplitude, the frequency of the mechanical signal can also play an important role in biological output. Previous work has shown that cyclic stretching of cells cultured on soft substrates induces spreading, stress fiber formation, and proliferation.^[^ ^46^ ^]^ Notably, several studies reported nuclear translocation of mechanosensitive proteins MRTF‐A and YAP under external mechanical loading while the duration, amplitude, and frequency of the chosen loading conditions varied.^[^ ^33^, ^38^, ^57^ ^]^ The rate at which forces are applied also influences force transmission and subsequent signaling.^[^ ^58^ ^]^ Our platform is capable of generating physiologically relevant dynamic mechanical signals and, in contrast to the existing platforms, is able to do so within a tunable engineered fibrous matrix. Finally, the rotation of the µ‐actuator could be dynamically modulated according to the real‐time mechanical feedback of cells informed by time‐lapse imaging. This novel experimental platform can in the future examine adaptive cellular force responses in homeostasis, pathology, and regeneration.

## 4 Experimental Section

### Fabrication of Fiber Networks

Suspended DexMA fiber matrices were fabricated through electrospinning and soft lithography as previously described.^[^ ^12^, ^13^ ^]^ Briefly, DexMA was dissolved at 0.5 g mL^−1^ in a 1:1 mixture of milli‐Q water and dimethylformamide with 1% w/v Irgacure 2959 photocrosslinker and 0.625 × 10^−3^ m methacrylated rhodamine (Polysciences, Inc., Warrington, PA, USA). For matrix displacement studies, 10% v/v blue carboxylate‐modified FluoSpheres (1.0 µm diameter, 2% w/v) was added to the solution. The electrospinning platform consists of a high‐voltage power supply (Gamma High Voltage Research, Ormond Beach, FL, USA), syringe pump (KD Scientific, Holliston, MA, USA), and a grounded copper collecting surface enclosed within an environmental chamber held at room temperature and 30% relative humidity (Terra Universal, Fullerton, CA, USA). DexMA electrospinning was conducted at a flow rate of 0.45 mL h^−1^, voltage of 7.0 kV, and gap distance of 6 cm. After electrospinning, fibers were stabilized by primary crosslinking under ultraviolet (UV) light (100 mW cm^−2^) for 60 s. To promote fiber–fiber welding, fiber networks were exposed to a humidified environment for 1 min. Fibers were collected on various poly(dimethylsiloxane) (PDMS; Dow Silicones Corporation, Midland, MI, USA) arrays of wells produced by soft lithography. Silicon wafer masters possessing SU‐8 photoresist (Microchem, Westborough, MA, USA) were first fabricated by standard photolithography. Briefly, a layer of SU‐8 2075 (100 µm thick) was spin‐coated on a 3 in. silicon wafer and patterned into arrays of various shaped wells spaced evenly within 12 × 12 mm squares. These masters were utilized to make PDMS stamps which were silanized with trichloro(1H,1H,2H,2H‐perfluorooctyl)silane and used to emboss uncured PDMS onto oxygen plasma‐treated coverslips. Resultant fiber‐well substrates were methacrylated by vapor‐phase silanization of 3‐(trimethoxysilyl)propyl methacrylate in a vacuum oven at 60 °C for at least 6 h to promote DexMA fiber adhesion to PDMS.

### Mechanical Characterization

To determine the tensile mechanical properties of individual fibers, three‐point bending tests were performed using a Nanosurf FlexBio atomic force microscope (AFM; Nanosurf, Liestal, Switzerland). Individual fibers were isolated on microfabricated PDMS troughs (200 µm tall and 200 µm wide) by brief (2–3 s) durations of electrospinning. Fibers were hydrated and deformed by an AFM tip (0.032 N m^−1^) loaded with a 35 µm diameter borosilicate glass bead positioned centrally along the fiber's length. Young's modulus was calculated from the resulting load‐displacement curves using known equations for a cylindrical rod undergoing three‐point bending with fixed boundaries.^[^ ^59^, ^60^ ^]^ To measure bulk mechanics of DexMA fibrous matrices, microindentation testing with a rigid cylinder was performed on a commercial CellScale Microsquisher (CellScale, Waterloo, ON, Canada). Cylinders (250 µm diameter, 200 µm tall) of SU‐8 photoresist were microfabricated and affixed to pure tungsten filaments (0.156 mm diameter, 59.6 mm length). Samples were indented to a depth of up to 35 µm at an indentation speed of 2 µm s^−1^.

### Assembly of Microactuators

Magnetic microactuators were fabricated from an electrodeposited nickel foil (Goodfellow, UK) using laser micromachining (DB Products, France). The nickel microactuators were dip‐coated with in uncured PDMS and cured at 60 °C for 6 h to protect actuators from oxidization and for ease of transport. PDMS‐coated nickel microstructures were transferred to the target locations on DexMA fiber networks using a custom‐made aspiration micropipette. Microcapillaries (CM Scientific, UK) were shaped using a micropipette puller (Sutter Instruments, USA) and microforge device (Narishige, USA). The tip of the micropipette has an inner diameter of 30 µm with a tilt angle of 25° to ensure application of firm suction to the microactuator (Figure S1, Supporting Information). The micropipette was connected to a microfluidic pressure pump (ElveFlow, France) for the pick‐up and release of the microactuator, and a piezoelectric xyz positioner (SmarAct, Germany), for control of the position of the pipette tip in 3D. After contacting the microactuator with the pipette tip, 700 mbar negative pressure was applied and maintained until translation to the target location where the microactuator was released with the application of 50 mbar positive pressure. Microinjection pipettes with an inner diameter of 5 µm at the tip were fabricated using the same approach (Figure S1, Supporting Information). A UV curable glue (Norland Products, USA) was injected around the microactuators using a pneumatic picopump (World Precision Instruments, USA) and the same xyz positioner. The injected glue around the laser cut nickel was locally polymerized using a digital micromirror device (Andor, Oxford Instruments, UK) connected to a UV light source (CoolLED, UK).

### Magnetic Control System

The circular magnetic control system consists of eight cube‐shaped NdFeB permanent magnets (HKCM Engineering, Germany) with a volume of 1 cm^3^ and flux density of 1.43 T. The magnetic field strength in the workspace was measured using a Hall sensor (Hirst, UK) that was attached to a piezoelectric xyz positioner (SmarAct, Germany). The array was attached to rotational piezo stage (Physik Instrumente, Germany) with 0.02° resolution via a 3D printed adapter (Formlabs, USA). A 3D printed environmental chamber was assembled on the stage for time‐lapse experiments. The motion of the stage was controlled using the GUI of a built‐in MATLAB program. The program allows programmable actuation modes including rotation to a given position with a set angular speed and oscillations around a given position over a user‐defined period.

### Topology Reconstruction using Image Processing

Fluorescence images of the fibers were taken using a laser scanning confocal microscope (LSM700 Upright Confocal Microscope, Zeiss Germany) to image rhodamine fluorescence at 555 nm. Microscopy images were binarized based on the intensity of pixels using a numerical implementation of Bresenham's line algorithm in MATLAB.^[^ ^61^ ^]^ Nodes and elements were converted into a sketch of lines using a CAD software (CATIA).

### Finite Element Implementation

Finite element simulations were performed using a commercial software (Abaqus) and the Explicit/Dynamic solver to resolve multi‐body contact between adjacent deforming fibers and rigid objects. The inputs to the computational model were the experimentally measured value of the Young's modulus of the fibers and the angular displacement of the microactuator. The fibers were modeled as linear elastic Timoshenko beam elements (B31) while accounting for finite deformations. Both the microactuator and the microindenter were defined as rigid shell elements with a reference point in the middle. The ratio of kinetic to total energy of the system was kept to less than 0.01 through mass scaling to ensure that simulations were performed in quasi‐static equilibrium. Fibers were discretized using a mesh that corresponds to beam elements of 4 µm in length.

### Fiber Network Deformation Tracking Via Fluorescent Beads

Fluorescence images of beads were acquired by Nikon Eclipse Ti inverted microscope with excitation at 365 nm wavelength during the actuation. TrackMate plugin of ImageJ was used to obtain the displacement data of every individual beads. For bead tracking, the Difference of Gaussian (DoG) detection algorithm was used with the estimated blob diameter of 4 µm and threshold of 3 µm. Displacement of all the particles in every frame were exported as a text file to link bead positions into tracks using a custom Matlab script.

### Cell Culture

NIH‐3T3 fibroblasts (Sigma‐Aldrich) and MDCK epithelial cells (Sigma‐Aldrich) were cultured in Dulbecco's modified Eagle's medium GlutaMAX (LifeTechnologies) supplemented with 10% fetal bovine serum (LifeTechnologies) and 1% penicillin‐streptomycin (LifeTechnologies). Cells were passaged every 2–3 days using Trypsin 0.25% EDTA (LifeTechnologies). Experiments were performed with cells that were conformed to be negative for mycoplasma.

### RGD Functionalization and Cell Seedings

DexMa fiber networks were functionalized with the integrin‐binding peptide CGRGDS (CPC Scientific, US) to enable cell adhesion, as described previously.^[^ ^12^ ^]^ Briefly, to couple the thiolated peptide to available methacrylates using Michael addition, dry peptide was dissolved in H_2_O containing 10 μg mL^−1^ phenol red (Sigma‐Aldrich) and 50 × 10^−3^ m HEPES (Sigma‐Aldrich). The solution was adjusted to pH ≈ 7.5 by the addition of NaOH (Sigma‐Aldrich). For functionalization of DexMA fiber networks, a final CGRGDS concentration of 2 × 10^−3^ m was used. The solution was transferred to the fiber networks and incubated for 45 min at room temperature. Following functionalization, substrates were rinsed with PBS prior to cell seeding. 200 000 cells were seeded on a single substrate, which was then incubated for 6 h before time‐lapse recording.

### Live Imaging

Cells were labeled with Hoechst 33342 (Thermo Fisher Scientific, Waltham, MA, USA) to visualize their nuclei during time‐lapse imaging. Phase‐contrast and fluorescence images were captured every 15 min for 6 h with an ORCA‐Flash4.0 digital CMOS camera (Hamamatsu, Japan) and a Plan Fluor 10× objective mounted on a fully motorized Nikon Ti Eclipse inverted microscope (Nikon Instruments, Japan). To visualize calcium signaling, cells were labelled with Calbryte 520 AM (AAT Bioquest). The working solution was composed of 4.5 × 10^−6^ m Calbryte in HHBS Hanks Buffer with HEPES (AAT Bioquest) supplemented with 0.04% Pluronic F127 (Sigma‐Aldrich). Cells on fiber networks were carefully washed with the HHBS Hanks Buffer prior to the addition of the dye solution. After 45 min incubation at 37 °C and 5% CO_2_, the dye was removed and devices were washed again with the buffer solution. Live imaging was performed in the buffer solution supplemented with HEPES. Movies were recorded at 100 Hz with a Plan Fluor 10× objective.

### Immunohistochemistry

DexMA fiber networks with cells were fixed with 4% formaldehyde and permeabilized with 0.2% Triton X‐100. Samples were subsequently incubated with Hoechst 33342 (Thermo Fisher Scientific, Waltham, MA, USA) and 488‐Phalloidin to visualize nuclei and F‐actin, respectively. Fluorescently labeled cells were imaged using an inverted confocal microscope (LSM 700; Zeiss, Oberkochen, Germany) equipped with a 20× objective.

### Statistics

Statistical significance was determined by unpaired *t*‐test with significance indicated by *p* < 0.05.

## Conflict of Interest

The authors declare no conflict of interest.

## Supporting information

Supporting Information

Supplemental Movie 1

Supplemental Movie 2

Supplemental Movie 3

Supplemental Movie 4

Supplemental Movie 5

Supplemental Movie 6

Supplemental Movie 7

Supplemental Movie 8

## Data Availability

The data that support the findings of this study are available from the corresponding author upon reasonable request.
