# Supplementary material for: Engineered Extracellular Matrices with Integrated Wireless Microactuators to Study Mechanobiology
Source: Adv Mater. 2021 Aug 7;33(40):2102641. doi: 10.1002/adma.202102641 (PMC11481068; doi:10.1002/adma.202102641)
Supplement: Supplementary file 1 — Supporting Information [file ADMA-33-2102641-s006.pdf]

© 2021 Wiley-VCH GmbH

ADVANCED MATERIALS

Supporting Information

for *Adv. Mater.,* DOI: 10.1002/adma.202102641

Engineered Extracellular Matrices with Integrated Wireless Microactuators to Study Mechanobiology

Fazil E. Uslu, Christopher D. Davidson, Erik Mailand, Nikolaos Bouklas, Brendon M. Baker, and Mahmut Selman Sakar**

Copyright WILEY-VCH Verlag GmbH & Co. KGaA, 69469 Weinheim, Germany, 2018.

Supporting Information

Engineered extracellular matrices with integrated wireless microactuators to study mechanobiology

Fazil E. Uslu, Christopher D. Davidson, Erik Mailand, Nikolaos Bouklas, Brendon M. Baker, Mahmut Selman Sakar**

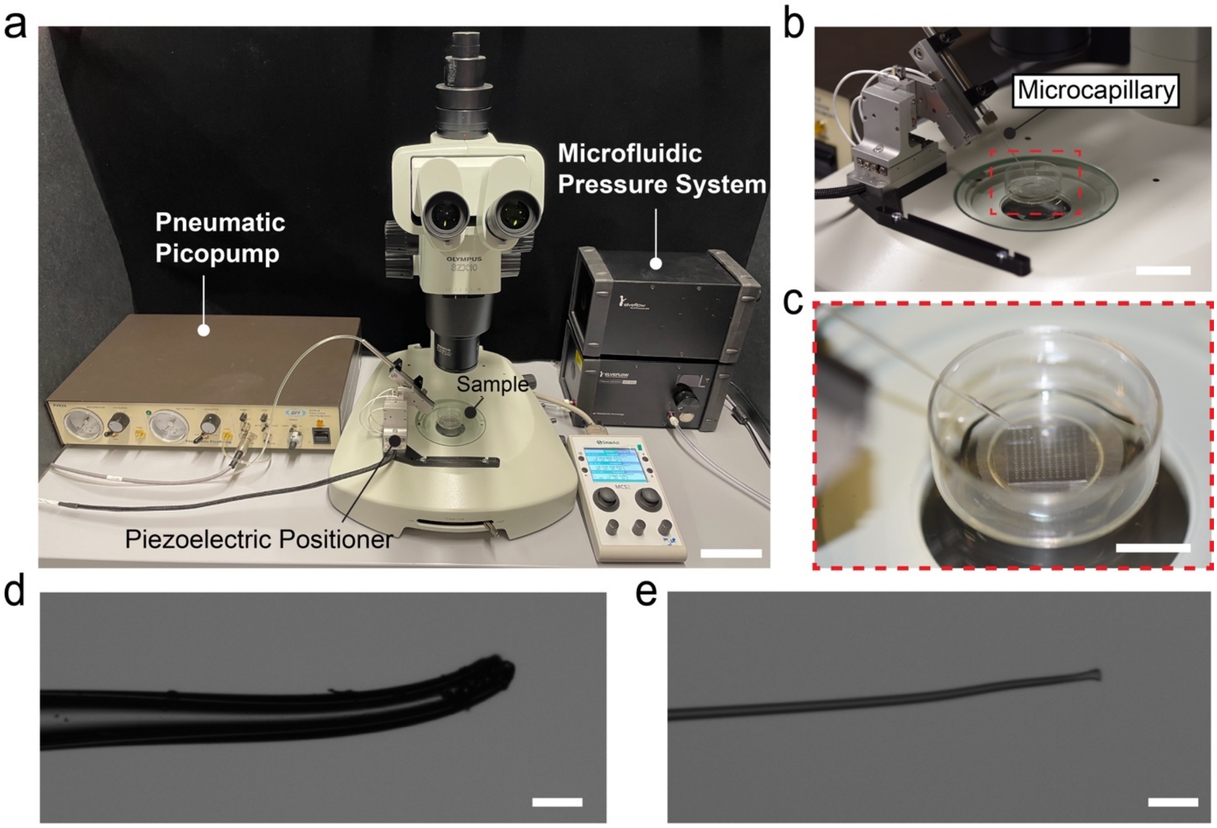

Figure S1 Robot-assisted microassembly of µ-actuators. a) A microfluidic pressure control system regulates pressure during the transport of the µ-actuator. A pneumatic picopump is used to controllably inject UV curable glue around the µ-actuator. The position of the capillaries are controlled by a 4-DOF piezoelectric positioner. Scale bar, 10 cm. b) A close-up view of the manipulator along with the capillary adapter. Scale bar, 3.5 cm. c) Wells containing fiber networks (n = 100) are within the yellow circle inside the Petri dish. Scale bar, 1 cm.

Microcapillaries that are pulled and forged for the (d) transport of μ-actuators and (e) injection of glue material. Scale bars, 50 μm.

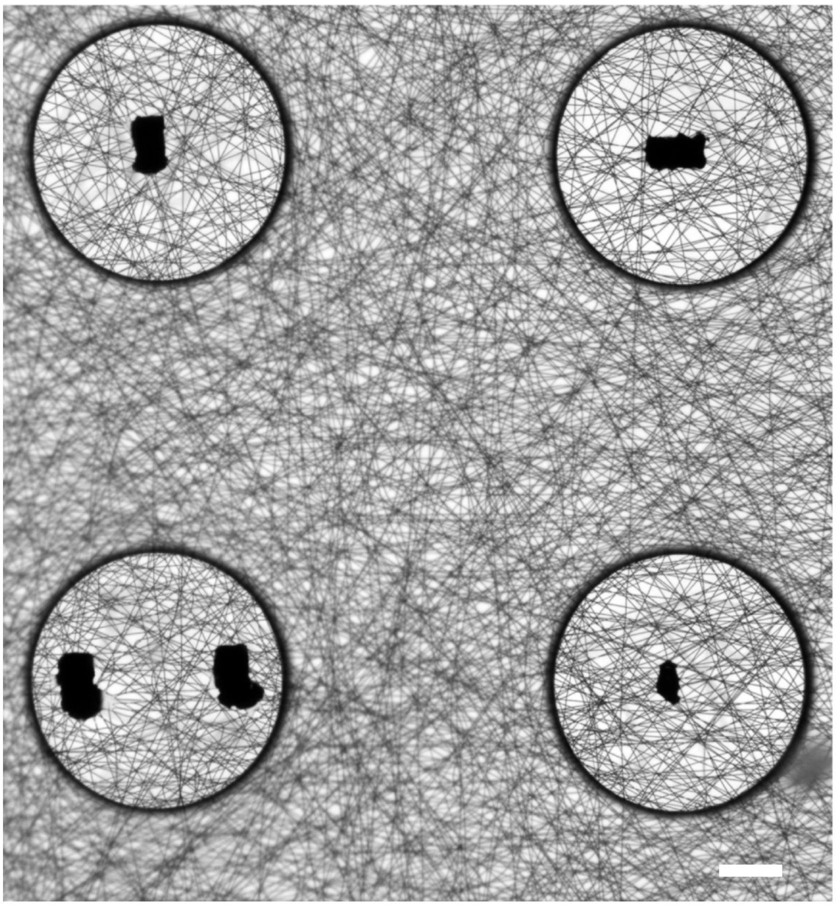

Figure S2 Fiber networks with various configurations of actuators. Single or multiple μ-actuators can be assembled onto the fibers at user-defined locations with desired orientations. The long side of the actuator can be as small as 50 μm. Scale bar, 100 μm.

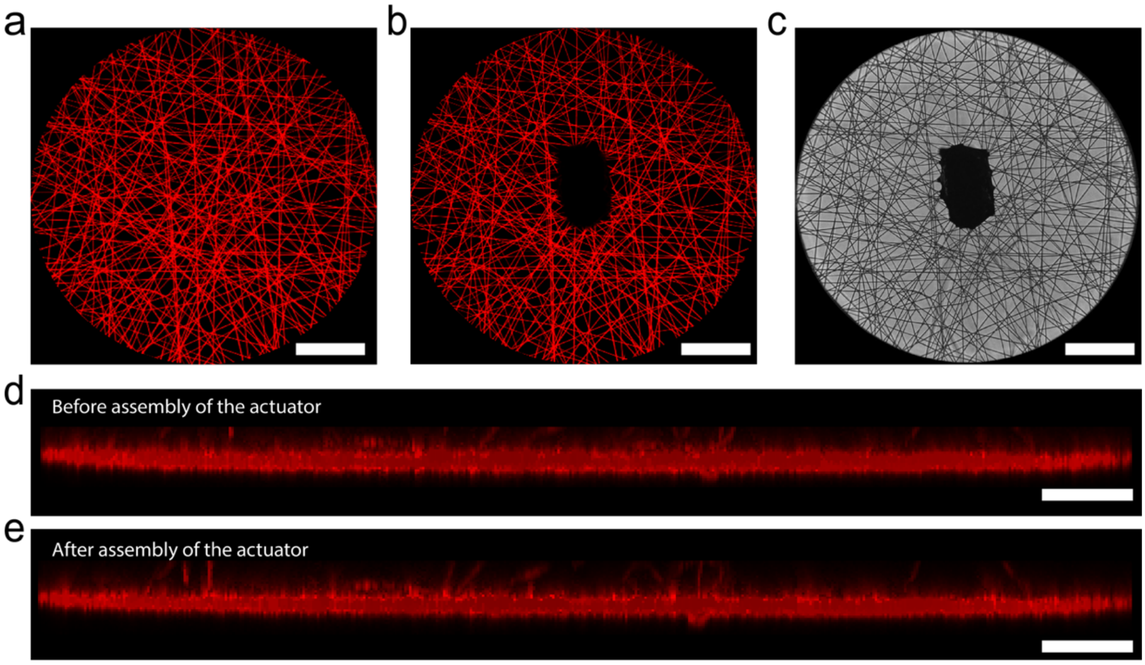

Figure S3 The effect of weight on the state of the fiber network. Top-down view of the fiber network before (a) and after (b) the assembly of the μ-actuator. The fibers were fluorescently labeled in red. Scale bar, 100 μm. c) Bright-field image of the fiber network after the assembly. Scale bar, 100 μm. Side view of the fiber network before (d) and after (e) the assembly of μ-actuator. Scale bar, 50 μm. Side view is generated by sectioning the 3D reconstruction of the fiber network, which is formed from the 2D confocal scans.

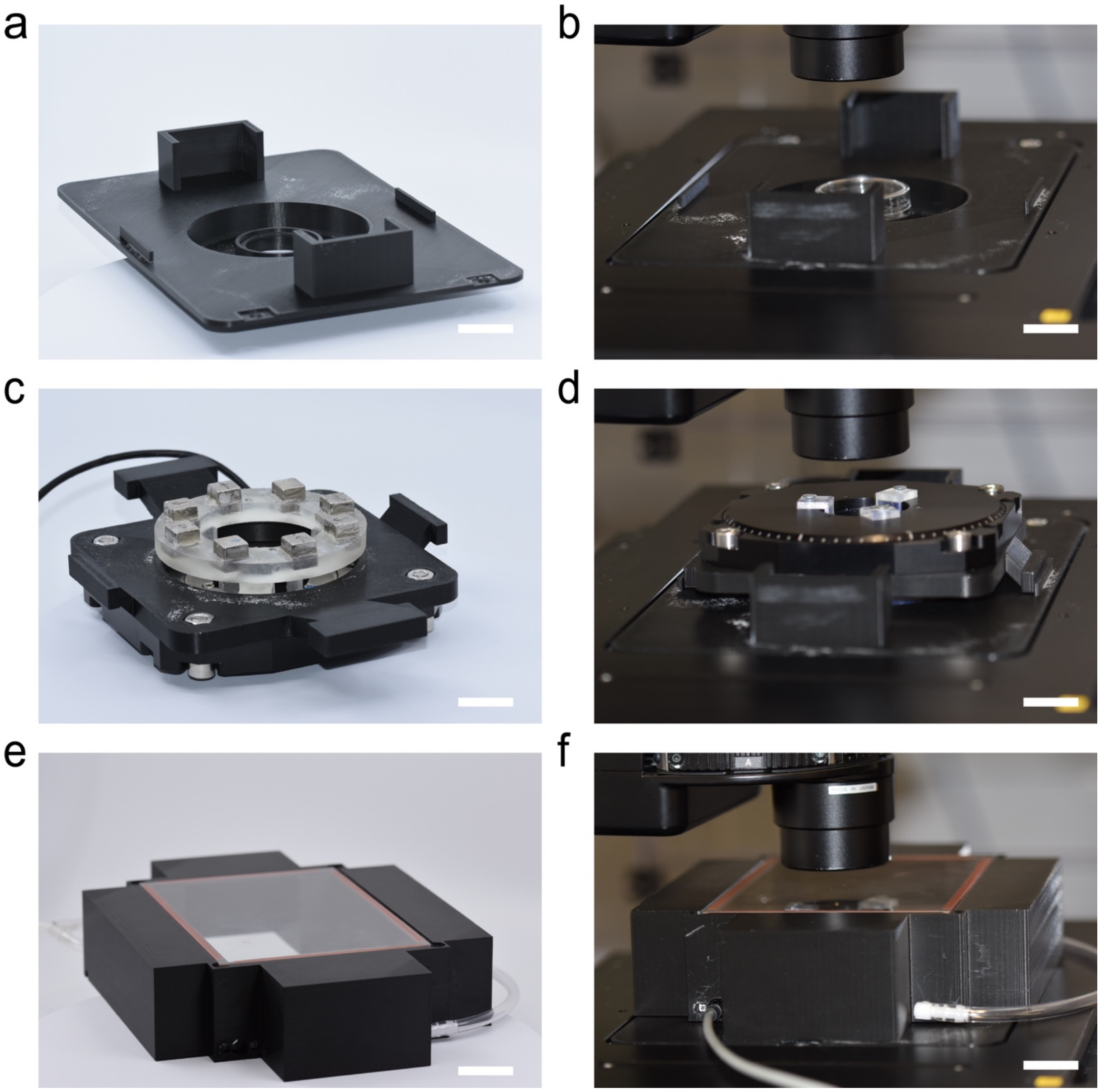

Figure S4 Magnetic manipulation system. a) Stage adapter part specifically designed for holding the magnet array on a motorized inverted microscope. b) The view of the adapter mounted on the microscope stage. c) The assembly of magnet array on the rotary motor. d) The nested manipulator secured on the microscope stage. e) Environmental chamber for maintaining physiological conditions during cell culture. f) Once the sample is placed in the middle of the manipulation platform, the environmental chamber seals the workspace. Scale bars, 2 cm.

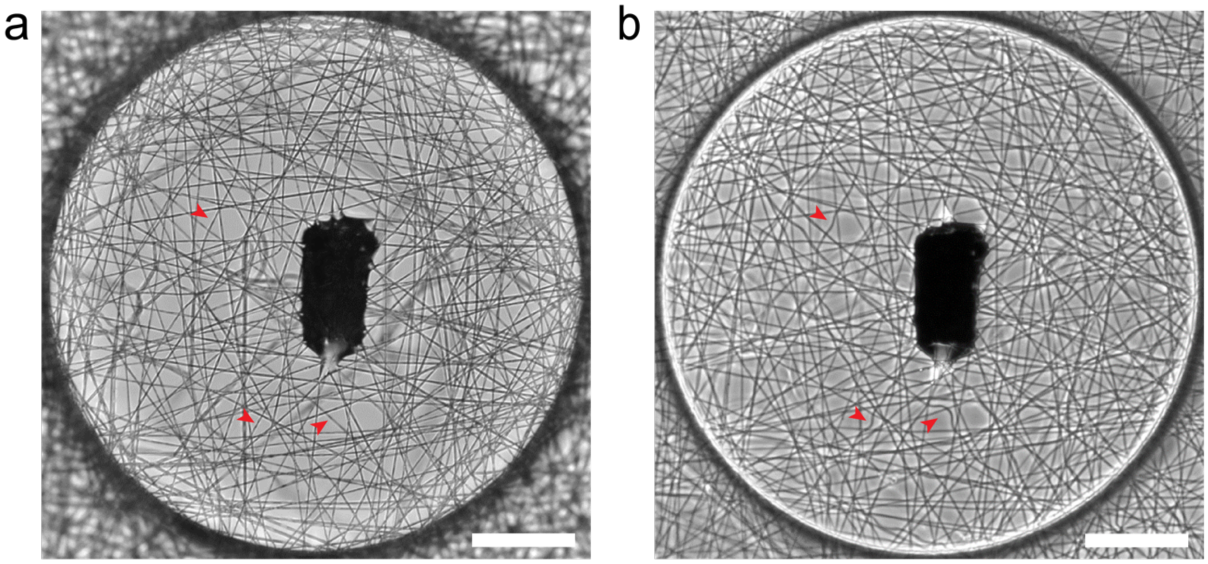

Figure S5 The effect of swelling and hydration on fiber mechanics. A fiber network in the (a) dry and (b) hydrated state. The red arrows point to three representative fibers where expansion with swelling leads to buckling. There are many more fibers in a given network that show mechanical instabilities after hydration. Scale bars, 100 µm.

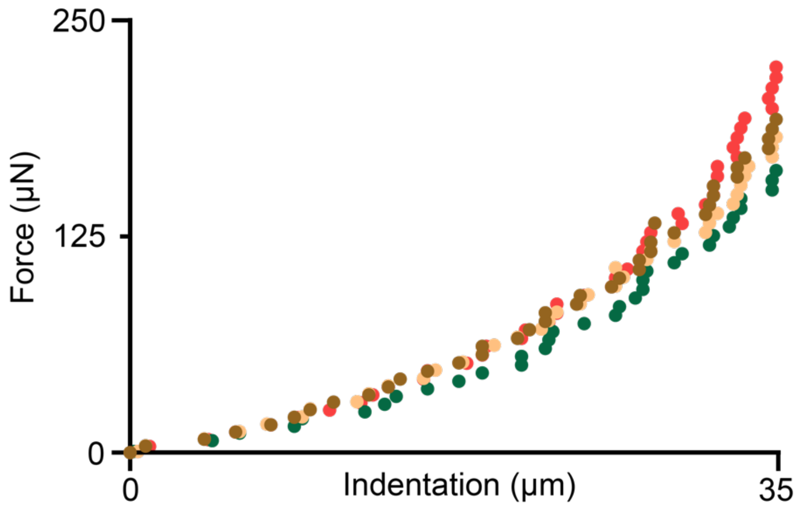

Figure S6 Indentation tests on fiber networks in air. Four different fiber networks are tested using a 250 µm indenter.

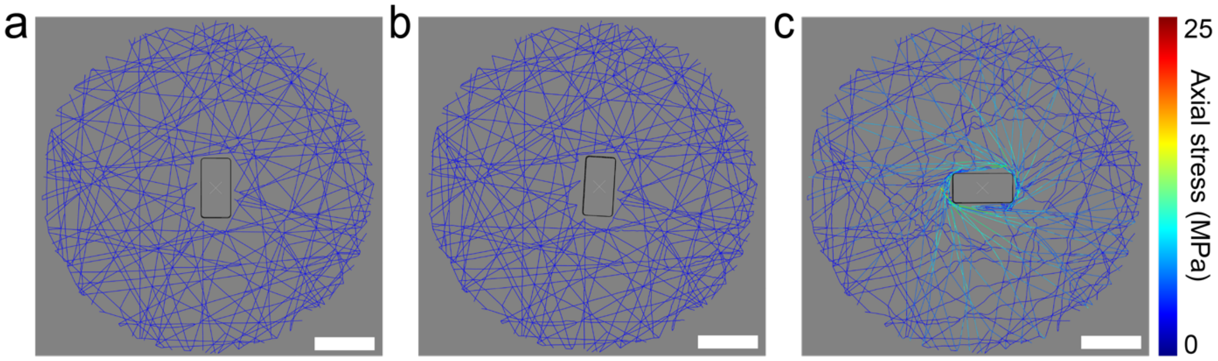

Figure S7 Simulated stress using magnetic torque as the input. a) Equilibrium configuration. b) The stress map upon application of $\tau = 1.06 \times 10^{-9}$ N·m. The μ-actuator rotates by 2.75º. c) The stress map upon the application of the scaled torque, $\tau = 3.15 \times 10^{-8}$ N·m. The μ-actuator rotates by 88º. Scale bars, 100 μm.

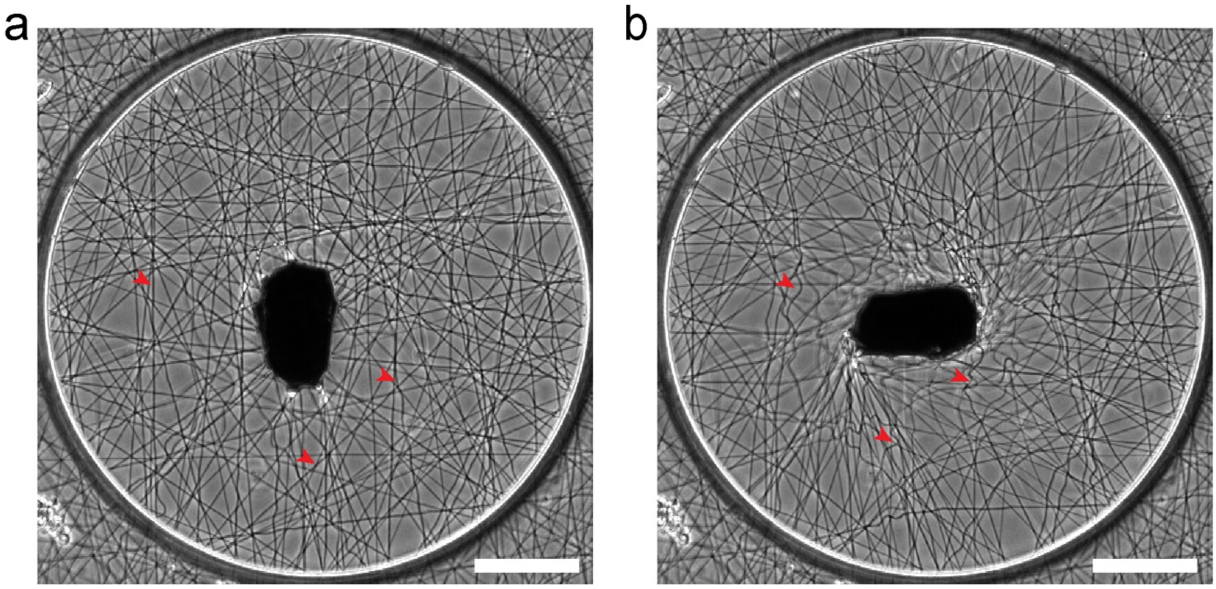

Figure S8 Buckling of fibers during actuation. a) A representative fiber network in the equilibrium configuration. b) Fiber network during the application of a magnetic torque that lead to 89º rotation of the μ-actuator. Red arrows point to fiber elements that display buckling upon actuation. Scale bars, 100 μm.

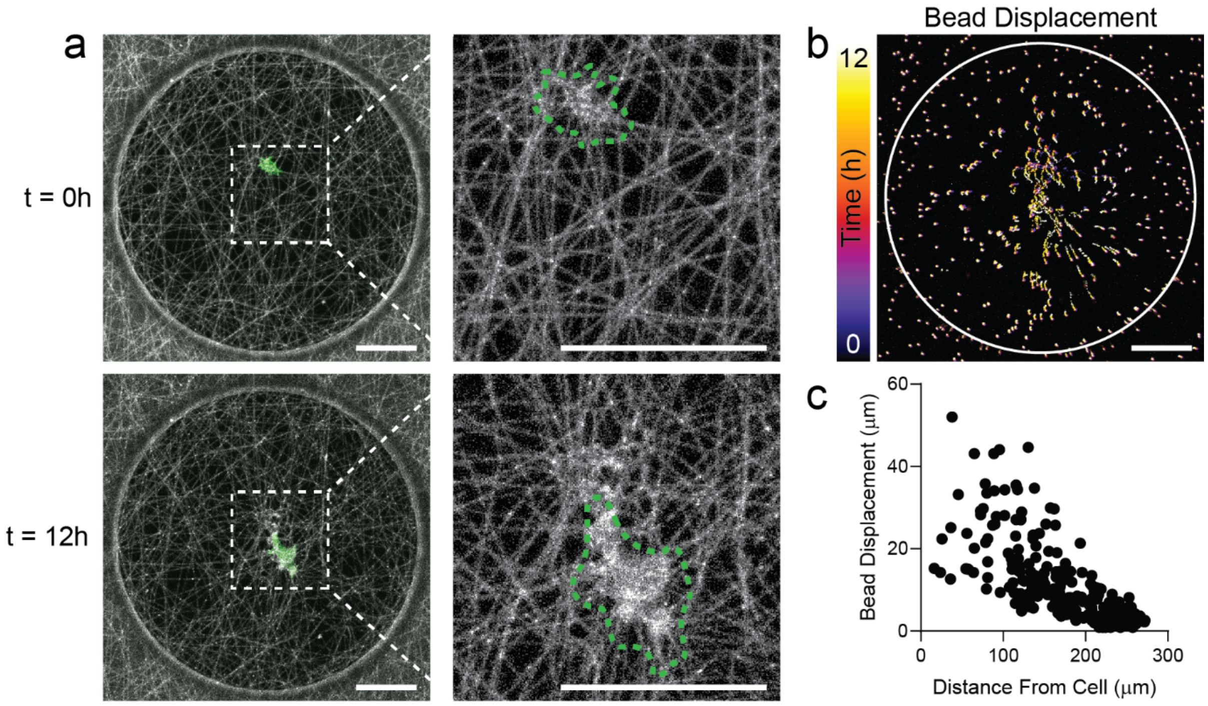

Figure S9 Cell force-mediated fiber reorganization. a) A single endothelial cell was seeded within a fiber network and imaged for 12 hours; F-actin(green), DexMA fibers (grey). Dashed boxes indicate locations of higher magnification images depicting fiber recruitment underneath the cell body (dashed green outline). b) Temporally color-coded overlay of fluorescent beads embedded within fibers over 12 hours following endothelial cell seeding and c) quantification of bead displacement distance as a function of its distance from the cell centroid. Scale bars, 100 μm.

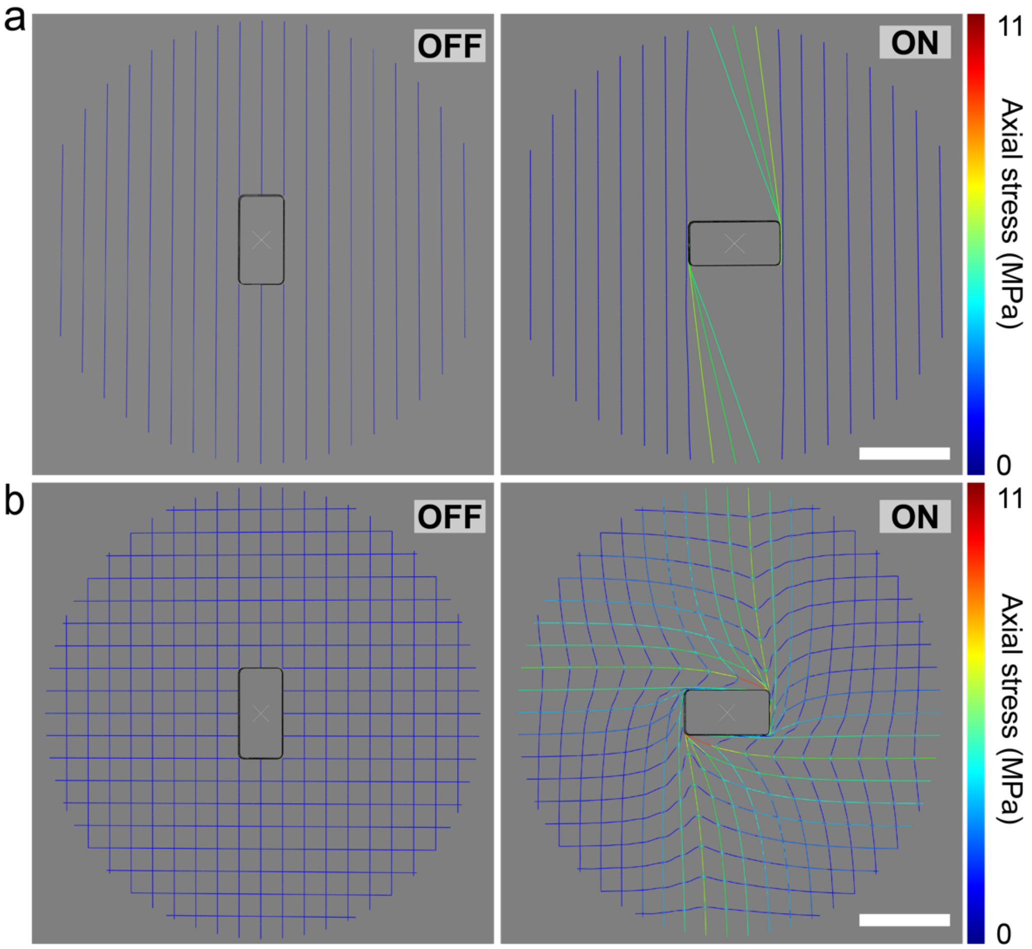

Figure S10 Computational analysis of the effect of fiber architecture on the distribution of stress. a) Fiber network with vertically aligned fibers before (left) and during (right) actuation. a) Fiber network with a regular grid architecture before (left) and during (right) actuation. The extreme example shows how certain areas of the network is not stimulated in the absence of proper connections among the fibers. Scale bars, 100 µm.

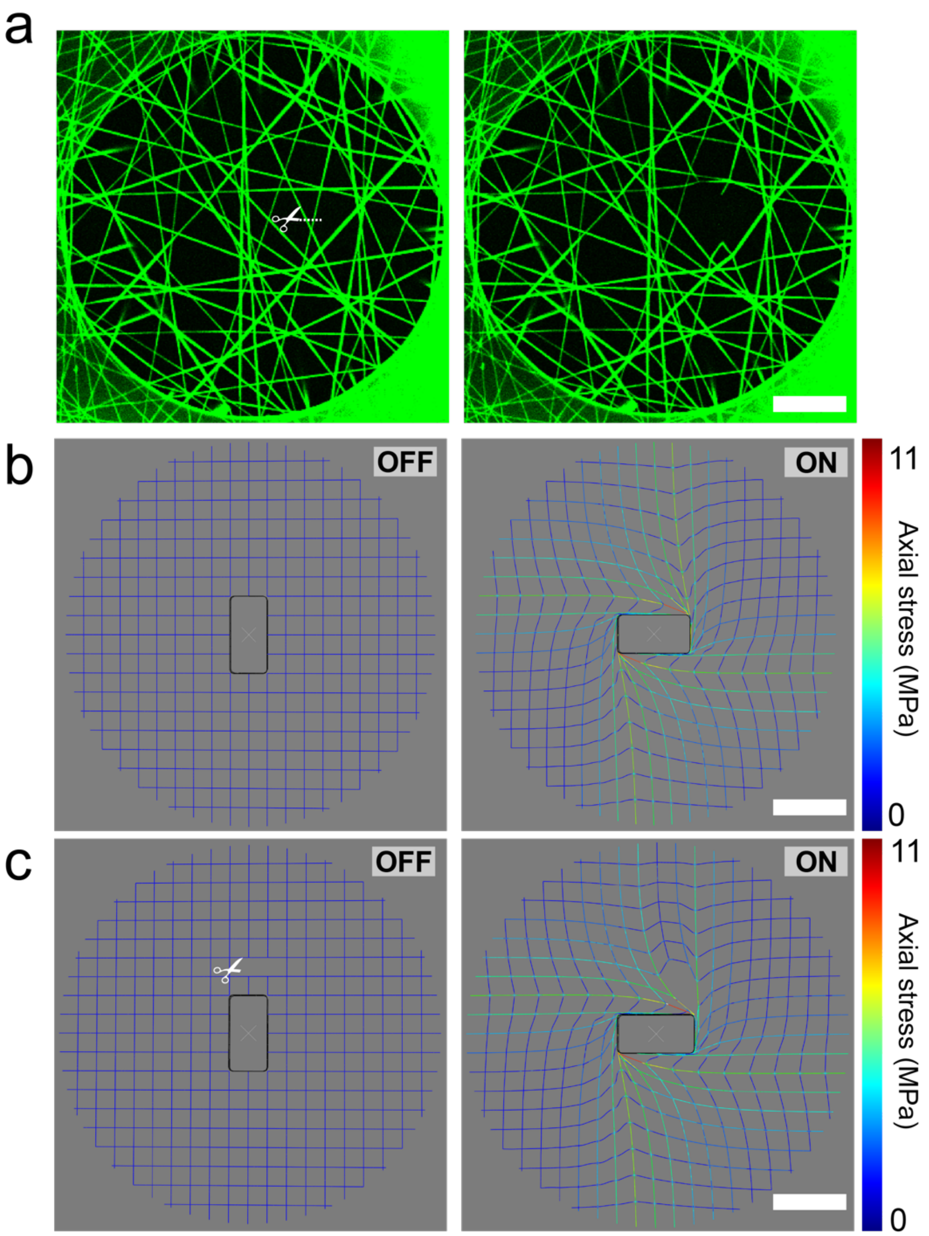

Figure S11 Laser microsurgery for subtractive manufacturing. a) Fluorescent images of a fiber network going through laser microsurgery. The scissor icon points to the disrupted link. Scale bar, 100 µm. b) The stress map on a fiber network with a regular grid architecture before and during actuation. (c) Removal of two links from the network shown in (b) leads to shielding of stress beneath the disrupted links. Scale bars, 100 µm.

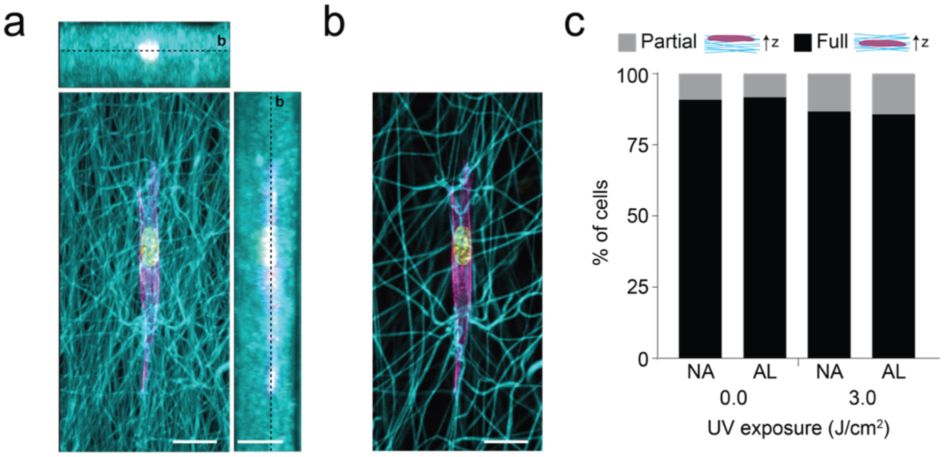

Figure S12 Dimensionality of DexMA fibrous matrices. Orthogonal x-y, x-z, and y-z maximum intensity projections (a) and single x-y plane (b) views of a composite confocal fluorescence image of representative NIH3T3 fibroblast within a suspended 3D fibrous matrix (rhodamine-labeled matrix fibers (cyan), F-actin (magenta), and nuclei (yellow); scale bars: 20 μm). (c) Percentage of cells that are partially or fully embedded within suspended fiber matrices 6 hours post seeding.

Table S1 Comparison of existing micromanipulation techniques with the presented technology.

Actuation Method	Substrate	Tethered (T) vs Untethered (U)	Local (L) vs Bulk (B) actuation	High-throughput	Long-term continuous actuation	Fiber recruitment
Mechanical probing with glass pipette [16, 20]	Glass slide	T	L	✗	✗	✗
AFM Indentation [21, 24, 27]	Glass slide	T	L	✗	✗	✗
Magnetic twisting cytometry [17, 25]	Well plate	U	L	✓	✗	✗
Optical tweezer [18]	Well plate	U	L	✗	✗	✗
Shear stress in channels [19]	Collagen gel	U	B	✓	✓	✗
Stretching substrate with servo motor [29, 32], pressure [33], dielectric elastomer [36]	elastomer	T	B	✓	✓	✗
Magnetic nanoparticles [26]	Glass slide	U	L	✓	✗	✗
Magnetic micropost [31, 35]	elastomer	U	L	✓	✓	✗
Light triggered thermomechanical actuation [34,37,38]	polymer coated glass	U	L	✗	✗	✗
Ferrofluid droplets [39-41],	Embryo	U	L	✓	✓	✗
Thermal actuation of hydrogel beads [42]	Spheroid	U	L	✓	✗	✗
**This Work**	**Engineered ECM**	U	L	✓	✓	✓

Supplementary Movies

Movie 1. The movie shows a fiber network that is mechanically loaded with a 250-μm indenter.

Movie 2. The movie shows a μ-actuator is rotated 90° clockwise with a speed of 0.09 rad/sec.

Movie 3. The movie shows a μ-actuator is rotated 90° clockwise with a speed of 9.425 rad/sec and oscillations with the same speed.

Movie 4. A movie showing the displacement of fluorescent beads during actuation of the fiber network.

Movie 5. Fibroblast interacting with the network and the μ-actuator at the equilibrium configuration. The magnetic field acts as a torque clamps and realigns the μ-actuator along the original axis.

Movie 6. Self-organization of directed migration initiated by the magnetic actuation.

Movie 7. A fibroblast performing slingshot migration.

Movie 8. Intracellular calcium signal recorded during actuation of an epithelial cell.
